# Supplementation with Low Doses of a Cod Protein Hydrolysate on Glucose Regulation and Lipid Metabolism in Adults with Metabolic Syndrome: A Randomized, Double-Blind Study

**DOI:** 10.3390/nu12071991

**Published:** 2020-07-04

**Authors:** Caroline Jensen, Hanna Fjeldheim Dale, Trygve Hausken, Jan Gunnar Hatlebakk, Ingeborg Brønstad, Gülen Arslan Lied, Dag Arne Lihaug Hoff

**Affiliations:** 1 Centre for Nutrition, Department of Clinical Medicine, University of Bergen, 5021 Bergen, Norway; hanna.dale@outlook.com (H.F.D.); trygve.hausken@helse-bergen.no (T.H.); jan.gunnar.hatlebakk@helse-bergen.no (J.G.H.); gulen.arslan@uib.no (G.A.L.); 2 Division of Gastroenterology, Department of Medicine, Haukeland University Hospital, 5021 Bergen, Norway; ingeborg.bronstad@helse-bergen.no; 3 National Centre of Functional Gastrointestinal Disorders, Haukeland University Hospital, 5021 Bergen, Norway; 4 Division of Gastroenterology, Department of Medicine, Ålesund Hospital, Møre and Romsdal Hospital Trust, 6026 Ålesund, Norway; dag.arne.lihaug.hoff@helse-mr.no; 5 Department of Clinical and Molecular Medicine, Faculty of Medicine and Health Sciences, Norwegian University of Science and Technology, 7491 Trondheim, Norway

**Keywords:** metabolic syndrome, cod protein hydrolysate, glucose metabolism, lipid metabolism, obesity

## Abstract

The risk of cardiovascular diseases and type 2 diabetes mellitus are increased in subjects with metabolic syndrome (MetS), and hydrolyzed fish protein may have favorable effects on metabolic health. Here, we investigated the effect of 8 weeks supplementation with 4 g of cod protein hydrolysate (CPH) on glucose metabolism, lipid profile and body composition in individuals with MetS in a double-blind, randomized intervention study with a parallel-group design. Subjects received a daily supplement of CPH (*n* = 15) or placebo (*n* = 15). Primary outcomes were serum fasting and postprandial glucose levels. Secondary outcomes were fasting and postprandial insulin and glucagon-like peptide 1 (GLP-1), fasting lipid concentrations and body composition. No difference was observed between CPH and placebo for insulin, glucose or GLP-1 after 8 weeks intervention. Fasting triacylglycerol decreased in both the CPH group and placebo group, with no change between groups. Fasting total cholesterol and low-density lipoprotein cholesterol decreased significantly within both groups from baseline to study end, but no difference was observed between the two groups. In conclusion, supplementing with a low dose of CPH in subjects with MetS for 8 weeks had no effect on fasting or postprandial levels of insulin, glucose or GLP-1, lipid profile or body composition.

## 1. Introduction

Hyperglycemia, hypertension, dyslipidemia and abdominal obesity form a cluster of interconnected metabolic abnormalities commonly known as the metabolic syndrome (MetS), which increases the risk of type 2 diabetes mellitus (T2DM) and cardiovascular diseases (CVDs) [1,2,3]. The prevalence of MetS varies depending on the definition used and the population studied, but it is estimated that between 20 and 30% of the adult population in most countries meet the diagnostic criteria for MetS [4]. Currently, the first-line therapy for MetS is education on lifestyle changes including physical activity and weight reduction, and improvement of risk factors closely linked to MetS [3]. The syndrome has significant negative impact on public health, and the rate of MetS is expected to continue to rise in adults and future generations unless we find effective strategies to prevent and reverse this development [4]. It is of interest to find ways to prevent and alleviate MetS, beyond the currently used strategies.

Based on associations seen between fish consumption and increased levels of high-density lipoprotein cholesterol (HDL-C) and reduced levels of triacylglycerols (TAG), increased consumption of fish may improve metabolic health and prevent development of MetS [5,6,7,8]. The nutrients in fish, such as iodine, vitamin D, taurine, long-chain omega-3 polyunsaturated fatty acids (n-3 PUFAs) and high-quality protein, may all contribute to the positive health effects of fish consumption [9]. There is also emerging evidence that proteins from fish contain bioactive peptides and may potentially modulate physiological processes in the human body, and contribute with a number of effects beyond their nutritional value as a source of energy and amino acids [10,11]. Bioactive peptides are released naturally by gastric digestion, produced by fermentation in the gut or through hydrolyzed protein added to the diet [12]. Animal and human studies suggest that hydrolyzed fish proteins given in low doses may have beneficial effects on lipid metabolism [13,14,15], postprandial glucose [14] and insulin regulation [16], as well as body composition and appetite [17,18]. Similar indications are observed in intervention studies with healthy overweight and obese adults given low doses (between 2.5 and 8 g) of supplements with unhydrolyzed cod protein [19,20,21].

We have previously investigated supplementation with low doses of a cod protein hydrolysate (CPH) on glucose metabolism and appetite in healthy adults [16,22], as well as supplementation for 6 weeks on inflammation and gastrointestinal health in patients with irritable bowel syndrome [23]. It is of interest to further evaluate the possible effects of low doses of CPH in a group of participants with metabolic abnormalities, such as subjects with MetS, over a longer period. Therefore, we aimed to investigate whether supplementation with low doses of 4 g of CPH per day for 8 weeks would have an effect on postprandial glucose metabolism and the appetite hormone glucagon-like peptide 1 (GLP-1), lipid profile and body composition in subjects with MetS. We hypothesized that the small peptides present in the CPH supplement would serve as rapidly absorbed bioactive peptides and lead to beneficial changes in glucose metabolism and an overall healthier metabolic profile.

## 2. Materials and Methods

### 2.1. Study Design

This study was as a multicenter, double-blinded, randomized, intervention with a parallel-group design. Participants with MetS received a daily supplement of 4 g of CPH (active ingredient) or placebo (no active ingredient) for 8 weeks. The primary outcome was fasting and postprandial glucose levels. Secondary outcomes were other metabolic and clinical parameters of the metabolic syndrome (waist circumference, fasting TAG and HDL-C), as well as fasting and postprandial insulin and GLP-1 levels, total cholesterol (total-C), fasting low-density lipoprotein cholesterol (LDL-C), and body composition. 

All subjects gave their written informed consent for inclusion before participation in this study. This study was conducted in accordance with the Declaration of Helsinki and all procedures involving human subjects were approved by the Regional Committee for Medical and Health Research Ethics of Central Norway (2018/2163). This study is registered at clinicaltrials.gov (NCT03807752).

### 2.2. Participants and Study Setting

Between March and September 2019, we recruited participants to this study through advertisement on the internal and external websites, on notice boards at Haukeland University Hospital (HUH), Bergen, and Ålesund Hospital, Ålesund, and at general practitioners in Bergen and the surrounding area. 

The criteria for inclusion were age between 40 and 70 years, body mass index (BMI) between 27 and 35 kg/m^2^ and the presence of MetS. Criteria for exclusion were chronic diseases or medication that were likely to interfere with the evaluation of the study endpoints (e.g., T2DM, medications known to affect glucose and lipid metabolism), allergy or intolerance to fish and/or shellfish, excessive alcohol consumption and/or drug as assessed by physician, acute infections or unwillingness to comply with the study requirements. As blood pressure was not an outcome measure, we allowed participants using certain types of blood pressure medications, not known to clinically affect glucose metabolism, to take part in this study. This included diuretics, calcium-channel blockers and agents acting on the renin-angiotensin system. Participants using beta-blocking agents or peripheral vasodilators were excluded. Four weeks prior to starting the intervention and during this study, the participants had to stop using any nutritional supplements with n-3 PUFAs. No changes in food consumption or level of physical activity were allowed. Lastly, the participants had to remain at a stable weight for the last three months and not be involved in any weight-loss programs prior to or during the intervention.

### 2.3. Definition of Metabolic Syndrome 

The Joint Interim Statement was used to define MetS, in which the presence of any three of five risk factors qualifies for a diagnosis of MetS: elevated fasting glucose, s-TAG, reduced HDL-C, increased waist circumference (WC) or elevated blood pressure [1]. Furthermore, we used the International Diabetes Federation cut-off points for central obesity (WC ≥ 80 cm in women; ≥94 cm in men) [24]. The cut-off points for the other components were as follows: s-glucose ≥ 5.5 mmol/L, s-TAG ≥ 1.7 mmol/L, s-HDL-C < 1.0 mmol/L in men and <1.3 mmol/L in women, systolic blood pressure ≥ 130 mmHg and/or diastolic blood pressure ≥ 85 mmHg [1].

### 2.4. Study Visits

All possible participants responding through the online recruitment form were pre-screened by telephone. Based on this, we invited potential participants to a screening visit to evaluate the presence of MetS and eligibility in terms of inclusion and exclusion criteria. The screening visit included a clinical examination by a physician, a review of medical history, vital sign (blood pressure, heart rate), anthropometric measures (weight, height and waist circumference) and blood sampling. We measured height and weight to the nearest 0.1 cm or 0.1 kg using an electronic weight/height scale and used these parameters to calculate BMI (kg/m^2^). Furthermore, at screening and end of study visit, we measured waist circumference (WC) according to WHO recommendations [25,26], i.e., locating the midpoint between the lower margin of the last palpable rib and the top of iliac crest, with arms relaxed at the side, at the end of a normal expiration and using a stretch-resistant measuring tape with constant tension.

On days of study visits, the participants came to the study center in the morning. After an overnight fast (i.e., after 9:00 pm the previous day, the participants could not eat/drink or use nicotine), all study procedures were performed. Fasting blood samples were taken, and anthropometric measures were performed. Body composition was measured by a bioelectrical impedance analysis (BIA) device at baseline and end of study visit (Body Composition Analyzer, BC-418 MA (model used in Ålesund), MC-180 MA (model used in Bergen), Tanita Corporation, Tokyo, Japan), following the manufacturers’ guidelines—barefoot with light clothing and an empty bladder.

Following the baseline measurement, the participants consumed a standardized breakfast meal (test meal). It consisted of two slices of semi-coarse bread (80 g bread, 50% whole wheat), 20 g white cheese, 25 g strawberry jam, 10 g margarine and 1.5 dL orange juice, providing a total of 1840 kJ (440 kcal), 69 g carbohydrate, 13.3 g protein and 14.3 g fat. We calculated the energy and macronutrient content using “*Kostholdsplanleggeren*” (Norwegian Food Safety Authority and The Norwegian Directorate of Health, Oslo, Norway) [27]. The participants consumed the meal within 15 min. Blood was drawn from an antecubital vein in the fasting state (−20 min), at 0 min, i.e., immediately after the meal was finished, and thereafter at 20, 40, 60, 80, 100 and 120 min. To induce an adequate blood glucose response, we calculated the required amount of energy and macronutrient in the test meal. We allowed free drinking of water, but did not serve coffee or tea.

The participants started the intervention on the day following the baseline visit. They opened the sealed bag with powder (active or placebo) and mixed well with 100 mL cold water. The participants consumed the supplement 10 min before breakfast every day for 8 weeks, except for the morning of the end of study visit due to required fast. 

### 2.5. Test Material

Firmenich Bjørge Biomarin AS (Ålesund, Norway) manufactured the test material. The flavored white powder came pre-packed in cardboard boxes from Pharmatech AS (Fredrikstad, Norway), with 56 sealed plastic-coated aluminum bags per box. The boxes were marked with A or B, i.e., blinded for both participants and personnel involved in this study. The intervention material contained 4 g of hydrolyzed protein from cod (cuttings and trimmings) in addition to 5 g glucose hydrate (Cargill), 2 g maltodextrin, 0.025 g tastegram powder flavor, 0.7 g citric acid and 0.1 g lemongrass durarome taste. The placebo contained 6.5 g of maltodextrin, 0.2 g citric acid, and was otherwise identical to the active material. The active material could not be identified from the placebo according to flavor or appearance.

The cod protein hydrolysate was produced by enzymatic hydrolysis of fresh frozen meat (cuttings and trimmings) from Atlantic cod (*Gadus morhua*). A batch of 500 kg frozen raw material was grounded, transferred to an incubator and mixed with sweet water at a ratio of 1:1, followed by stirring at 80 rpm and heating to 55 °C. The enzyme preparation Protamex^®^ (Novozymes AS, Copenhagen, Denmark) was then added, following incubation for 45 min, at 55 °C and pH 7.0. The preparation (incubate) was heated to 90 °C for 15 min to inactivate the enzyme. The enzyme-inactivated incubate was passed through a rotating sieve (Swenco, Sweden) to remove any bone fragments. A two-phase centrifugation (Alfa Laval AS, Denmark) was used to separate the peptide-containing water-soluble fraction (the hydrolysate) from the indigested residue. This was followed by ultrafiltration and dehydration of the soluble phase to a 50% dry matter concentrate, which was spray dried to a powder. The spray dried CPH powder contained 89% crude protein, 0% carbohydrate, <0.2% fat, 0% carbohydrate, <3.0% water, 0.1% sodium chloride, 1.7% sodium, 0.07% chloride and 10% ash. Of the total amino acid content in the hydrolysate, the free amino acids constituted 4.77%, and the ratio between essential amino acids and non-essential amino acids was 0.70. When analyzing the molecular weight (MW) of the hydrolysate, approximately 90% of the peptides present in the hydrolysate had a MW of 2000 Daltons (Da) or less, which corresponds to peptides consisting of 18 amino acids or less. Furthermore, approximately 75% had a MW of 1000 Da or below, corresponding to 10 amino acids or less, and 55% had a MW of 500 Da, corresponding to 5 amino acids or less. Approximately 25–30% of the hydrolysate was small dipeptides and free amino acids, with a MW of less than 200 Da. The composition of amino acids and taurine content of the spray dried CPH powder are given in a previous publication [16].

### 2.6. Estimation of Energy and Macronutrient Intake

To determine individual diet habits, the participants recorded food and drink intake in a three-day prospective food diary, including one weekend day, before the baseline visit and before the end of study visit. Energy and protein intake from the supplement was added to the end of study dietary records (CPH group: 44 kcal, 4 g protein; placebo group: 46.5 kcal, 0 g protein). We used dietary records to evaluate whether any changes were made in the participants diets during the intervention period and to record diet patterns. Calculations of energy and macronutrient intake were determined using “*Kostholdsplanleggeren*” [27]. 

### 2.7. Analyses of Blood Samples 

Samples for safety purposes (albumin, prealbumin, leucocytes, thrombocytes, hemoglobin, sodium, potassium, alanine aminotransferase, alkaline phosphatase, creatinine and aspartate aminotransferase) were taken at the screening and end of study visit. Glycated hemoglobin (HbA1c), total-C, TAG, LDL-C, HDL-C and fasting and postprandial serum insulin and glucose were measured at baseline and end of study visit. All tests were analyzed according to standard accredited methods at the routine hospital laboratories (Department of Medical Biochemistry and Pharmacology, HUH, and Department of Medical Biochemistry, Ålesund Hospital). 

Samples for GLP-1 determination were collected in Vacuette^®^ EDTA-K2 tubes, ref#454047 (Greiner Bio-One GmbH, Frickenhausen, Germany), with 20 μL dipeptidyl peptidase-4 inhibitor (DPP4-010; DRG Diagnostics, Marburg, Germany) added prior to sampling. Plasma for fasting and postprandial GLP-1 at baseline and end of study was obtained by centrifugation of EDTA blood at 1800× *g* and −4 °C for 10 min, within 20 min after blood sampling. Plasma GLP-1 was analyzed by ELISA method (GLP-1, Active form (High Sensitivity ELISA), Code No. 27700, (Immuno-Biological Laboratories Co., Ltd., IBL Japan)).

### 2.8. Randomization

To allocate the participants, we used a web-based data collection and randomization system developed and administered by the Norwegian University of Science and Technology, Trondheim, Norway. We used block randomization to create the random assignment order and stratified for center (Ålesund or Bergen). A person with no practical involvement in the trial coded the test materials. The participants, study investigator, and all other personnel involved in this study were blinded to group allocation. Study investigators were given the key of randomization after the trial was finished and the statistical analyses were carried out. 

### 2.9. Statistical Analyses 

We performed statistical analyses by IBM SPSS Statistics, Version 26.0 (IBM Corp., Armonk, NY, USA) and GraphPad Prism version 8.4.1 (GraphPad Software, Inc., San Diego, CA, USA. The latter was used for graphical work. The data is presented as the mean ± SD, unless otherwise stated. Normality was evaluated by the Shapiro–Wilk test and histograms, and non-normally distributed data was log-transformed before using parametric statistical tests. We tested changes within groups from baseline to end of study by the paired sampled *t*-test, and changes between groups by the independent samples *t*-test. We examined group differences over time for continuous outcome (postprandial measurements of insulin, glucose and GLP-1) in a linear mixed-effects model with repeated measures. The trapezoid rule was used when calculating the area under the curve (AUC) using only end of study day, comparing the active and placebo group. Level of significance was set to *p* < 0.05. Two participants in the placebo group were excluded from the statistical analysis of lipid parameters (TAG, total-C, LDL-C and HDL-C), due to the use of lipid-lowering drugs (Simvastatin (Zocor), Atorvastatin).

To our knowledge, the possible effects of supplementation with a hydrolyzed cod protein have not been investigated previously in overweight and obese subjects with MetS. Due to a lack of similar studies, a power calculation was not performed. According to the protocol, we planned to recruit 60 participants in this study, with 30 participants in each group (a minimum of 20 in each group). This is a number similar to what has been previously reported in studies with supplementation of low doses of cod protein in humans [20,21]. We did not reach the target population due to difficulties with the recruitment of eligible participants. Participants were recruited between April and September 2019, but due to time constraints and limited resources, the inclusion of new participants had to stop in September. 

## 3. Results

### 3.1. Participants 

**Figure 1:**
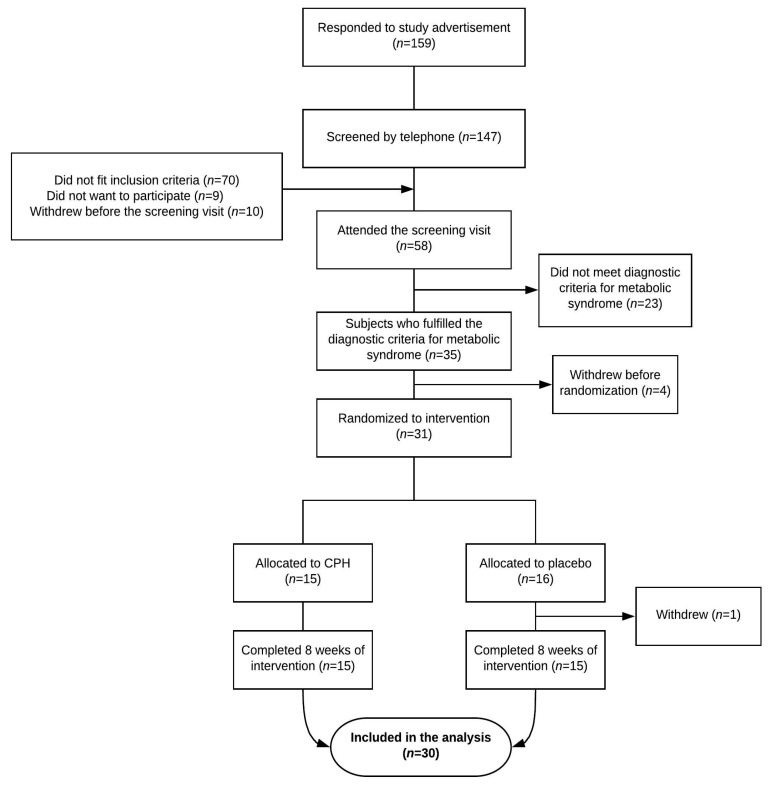
Overview of the participant flow during this study.

Of 58 participants attending the screening visit, 35 subjects had MetS and could be included in the trial. Four participants withdrew before randomization. Thirty participants completed the intervention according to study protocol and were included in the statistical analysis (Figure 1). At the screening visit, the groups were comparable (Table 1). 

### 3.2. Estimated Intake of Energy and Macronutrient 

The estimated intake of energy and macronutrients calculated from the dietary records did not differ between the groups at baseline, and we did not observe any changes within or between groups during the course of this study (Table 2).

### 3.3. Anthropometric Measurements 

Body weight (kg), fat mass (kg, %), fat-free mass (kg), BMI (kg/m^2^) or total body water (kg) did not differ within or between the groups (Table 3). Waist circumference increased within both the CPH group and the placebo group, but no differences were observed when comparing the two groups (Table 3).

### 3.4. Glucose Homeostasis

**Figure 2:**
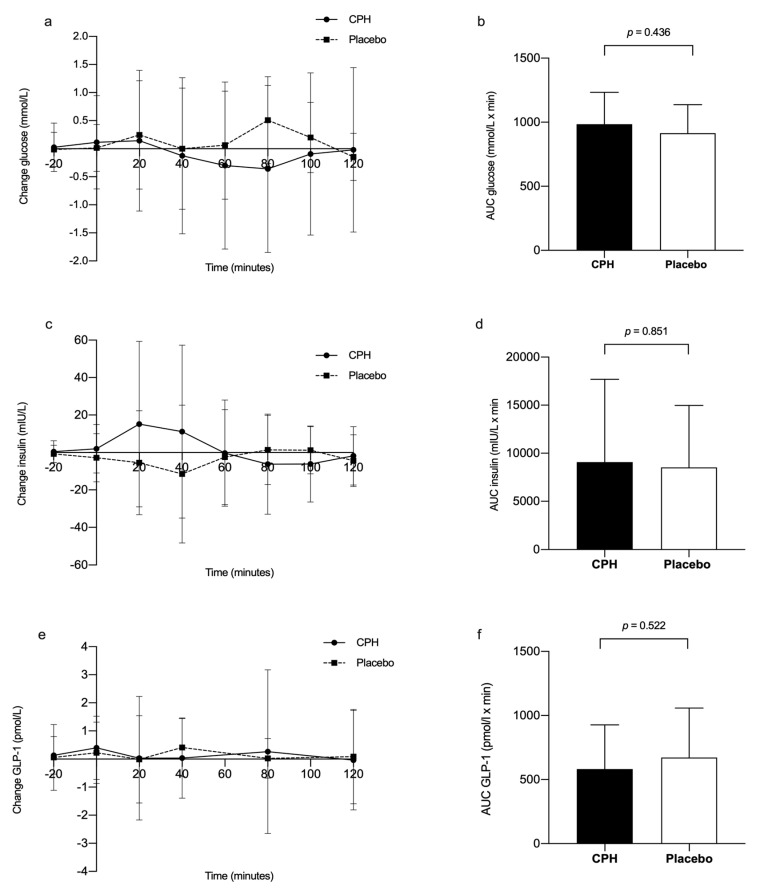
Glucose, insulin and glucagon-like peptide 1 (GLP-1) response in subjects with metabolic syndrome supplemented with cod protein hydrolysate (CPH) (*n* = 15) or placebo (*n* = 15) for 8 weeks. Change (8 weeks – baseline) in (**a**) serum glucose, (**c**) insulin and (**e**) plasma GLP-1 after a standardized breakfast meal, comparing the CPH group (solid line) with the placebo group (stippled line). The first postprandial blood samples (= 0 min) were taken 15 min after the test meal was served. For two individuals in the placebo group, only the fasting levels are included in the graphs. The area under the glucose curve (**b**, AUC glucose), insulin curve (**d**, AUC insulin) and GLP-1 curve (**f**, AUC GLP-1) for the CPH group (black bar) vs. the placebo group (white bar) at end of study. Values are presented as the mean ± SD.

Adjusted for time and visit, the glucose levels were on average 0.55 mmol/L higher for CPH compared to placebo, but the linear mixed-effects model with repeated measures did not reveal any significant differences between the groups (95% CI: (−0.44, 1.53), *p* = 0.267). We observed no change in glucose levels from baseline visit to end of study visit in either of the two groups (overall change: −0.014 mmol/L, 95% CI: (−0.19, 0.16), *p* = 0.876) (Figure 2a). Similarly, no difference in insulin levels was observed between the two groups after 8 weeks intervention. The insulin levels were on average 0.63 mIU/L higher for CPH compared to placebo (95% CI: (−31.32, 32.58), *p* = 0.968), and we did not observe any changes in insulin levels from baseline visit to end of study in either of the groups (overall change: 0.57 mIU/L, 95% CI: (−4.79, 5.92), *p* = 0.836) (Figure 2c). Furthermore, no difference in fasting or postprandial GLP-1 levels was observed between the two groups. The GLP-1 levels were on average 0.83 pmol/L higher (back transformed estimate) for participants who received CPH compared to placebo, but this was not significantly different (95% CI: (0.61, 1.13), *p* = 0.221). We did not observe any changes in GLP-1 levels from baseline visit to end of study visit in either of the groups (overall change: 0.95 pmol/L, 95% CI: (0.90, 1.01), *p* = 0.079) (Figure 2e). 

We observed no significant interactions between group and visit (baseline vs. end of study), group and time, time and visit or between group, visit and time for insulin, glucose or GLP-1. The AUC calculated from the fasting through the postprandial test points did not reveal any significant differences in insulin, glucose or GLP-1 levels between the CPH group and placebo group after 8 weeks (Figure 2b, d and f, respectively). 

At baseline, HbA1c was on average 37.5 ± 4.47 mmol/mol in the CPH group and 35.7 ± 3.40 mmol/mol in the placebo group, with no differences between groups (mean diff: 1.87, 95% CI: (−1.102, 4.835), *p* = 0.208). HbA1c did not change during the supplementation period within the CPH group (mean diff: 0.40 mmol/mol, 95% CI: (−1.15, 1.95), *p* = 0.589) or the placebo group (mean diff: 0.00, 95% CI: (−1.24, 1.24), *p* = 1), and there was no difference between the groups (mean difference: 0.40, 95% CI: (−1.49, 2.29), *p* = 0.669). 

### 3.5. Lipid Parameters 

**Figure 3:**
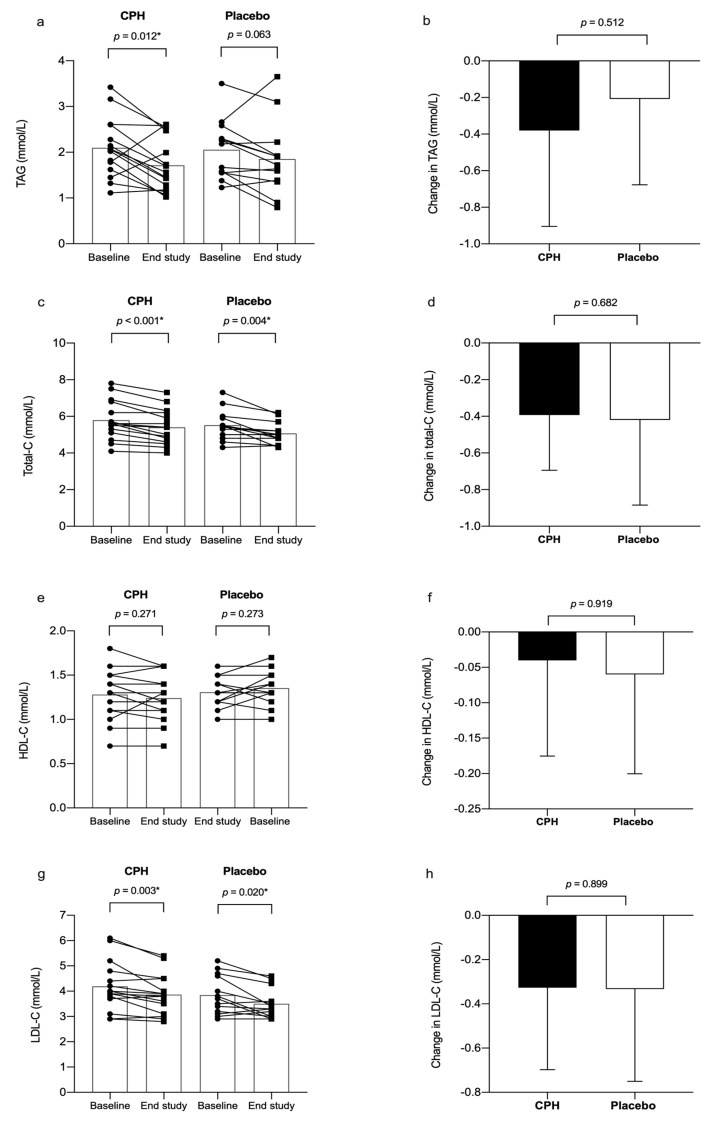
Fasting and change in serum levels of triacylglycerol (TAG) (**a**,**b**), total cholesterol (total-C) (**c**,**d**), high-density lipoprotein cholesterol (HDL-C) (**e**,**f**) and low-density lipoprotein cholesterol (LDL-C) (**g**,**h**) in subjects with MetS after 8 weeks intervention with cod protein hydrolysate (CPH) (*n* = 15) or placebo (*n* = 13). Two subjects from the placebo group were excluded from the statistical analyses of lipid parameter due to the use of lipid-lowering drugs. A (**c**,**e**,**g**), results are presented as individual graphs with fasting levels at baseline and end of study for each subject, with bars showing the mean in each group. *p*-values were calculated from a paired samples *t*-test. B (**d**,**f**,**h**), changes were calculated as individual end of study values minus baseline values for each of the indicators. Results are presented as the mean difference. *p*-values were calculated using the independent samples *t*-test. Significant *p*-values are marked with asterisk (*).

At baseline, no differences between the groups were observed for fasting TAG, HDL-C, LDL-C, total-C or total-C: HDL-C ratio. Fasting total-C and LDL-C were significantly decreased within both groups after 8 weeks of intervention, with no differences between the groups (Figure 3). Fasting TAG was reduced within the CPH group (mean diff: −0.81 mmol/L (back transformed estimate), 95% CI: (0.694, 0.948), *p* = 0.012), but did not differ from placebo (Figure 3). We did not observe any changes within or between groups for for fasting HDL-C and total-C: HDL-C ratio during the course of the study (Figure 3).

### 3.6. Adverse Effects 

Biochemistry for safety purposes were all within normal range. Seven participants reported discomfort during the intervention period—four in the CPH group and three in the placebo group. One participant in the CPH group reported that the supplement tasted bad and caused retching. Two participants in the CPH group reported heartburn, and two reported nausea at the beginning of the intervention period, but this was transient. In the placebo group, one participant reported myalgia, one reported itchy rash in the face and one reported nausea, but all three participants were unsure whether these experiences were related to the intervention. 

## 4. Discussion

The main aim of this study was to investigate whether daily supplementation with low doses of CPH for 8 weeks would have effect on fasting and postprandial glucose levels in participants with MetS. We hypothesized that supplementation with CPH would lead to beneficial changes in glucose metabolism and an overall healthier metabolic profile. We did not observe any significant effects of the supplement on the primary outcome measure (fasting and postprandial glucose levels) when compared to placebo. Furthermore, we found no effect on fasting or postprandial insulin or GLP-1 levels, lipid parameters or body composition after 8 weeks supplementation with CPH. 

Previous human intervention studies have reported improvements in postprandial glucose and insulin levels after supplementation with fish protein [20,28], and similar findings have been observed in animal studies [14,29,30]. Furthermore, we have previously shown that the postprandial insulin concentration in serum was significantly lower in normal-weight adults given one single dose of CPH compared to control (unhydrolyzed casein), without an effect on glucose levels [31]. Considering that the subjects in the current trial had MetS, the lack of effect is surprising, but there could be several reasons for this. Firstly, we gave the participants a fixed dose of 4 g of CPH, whereas we used weight-adjusted doses of CPH in previous studies, to reduce the effect of body weight variations [16,31]. In the current trial, the participants in the CPH group had a body weight ranging from 77 to 133 kg, meaning variation from 52 to 30 mg/kg body weight of CPH per day, which could possibly affect the overall results. It was not feasible to weight-adjust the doses in the current trial, and we therefore chose to use a fixed dose similar to what has been used in other studies and based on results from a previous study conducted by our research group [20,21,31]. Secondly, the participants in the current trial only received a daily morning dose of CPH. Previous studies have suggested that distributing the doses throughout the day might lead to a more potent effect due to a constant flow of bioactive peptides in circulation [32,33].

The estimated average daily protein intake for participants in the CPH group was 85 g/day, and only 4.7% of the total daily protein intake came from the supplement. Therefore, we do not presume the protein intake as such to cause any effect on postprandial glucose regulation and lipid profile. We believe that an effect may be due to particular peptide sequences in the supplement. Bioactive peptide sequences have been identified in other fish protein hydrolysates [34]. We did not test for the presence of such sequences in this particular study, which would have strengthened the design. 

Dietary proteins play a role in the regulation of lipid metabolism, and beyond the quantity of protein, the composition of amino acids and bioactive peptides are suggested to be of importance [35]. Associations between a high intake of lean fish and reduced levels of serum TAG [5,8], as well as increased levels of HDL-C [8], have been reported in previous cross-sectional studies. Beneficial effects have also been reported from intervention studies in animals and humans [36,37,38]. In the current trial, we observed a significant reduction in serum fasting TAG levels in the CPH group, with a decline of 18% from baseline to end of study. Considering that a weight reduction of 5–10% has been shown to cause a 20–30% reduction in TAG levels, whereas general improvement of nutrition-related practices can lead to a TAG-lowering effect of between 20 and 50% [39], a reduction of 18% in TAG levels after 8 weeks of supplementation with CPH, with no change in body weight or composition, is interesting. However, these changes did not differ from the placebo group, hence the results should be interpreted with caution. In line with findings from previous intervention studies with low doses of cod protein in healthy, overweight adults [20] and normal-weight adults [33], we observed reduced levels of LDL-C within the CPH group. However, a reduction in LDL-C was also observed within the placebo group, and no differences were found when comparing the two groups. Overall, we did not observe any effects of 8 weeks CPH supplementation on lipid parameters.

Proteins are considered to be the most satiating of the macronutrients [40], and there are indications that fish protein, compared to other animal proteins like beef and chicken, have a greater effect on satiety [41]. The gut hormone, GLP-1, released in response to intake of food from the enteroendocrine L cells, is involved in appetite regulation and contributes to glycemic control by slowing gastric emptying, stimulating insulin secretion and suppressing the secretion of glucagon [42,43,44]. A previous study in overweight individuals found increased levels of GLP-1 after 3 months supplementation with 1.4 and 2.8 g of blue whiting hydrolysate, when compared to placebo (whey protein isolate) [17]. In the current trial, we did not see any effect of supplementation with 4 g of CPH for 8 weeks on fasting or postprandial GLP-1 levels. In the trial by Nobile et al. [17], all groups had a caloric restriction of −300 kcal per day, which may have influenced the result and might suggest that a reduction in calories in combination with supplementation of fish protein hydrolysate is a more effective approach than supplementation alone. 

There are some limitations to the present study. Firstly, the lack of a power analysis and the small number of subjects per group might explain why we were not able to observe any effect of the intervention. Secondly, we did not reach the target population according to protocol, due to difficulties with recruiting eligible subjects and strict inclusion and exclusion criteria. We had to stop the inclusion period due to time constraints and limited resources, and the low number of subjects might have compromised this study. By choosing a cross-over design, we could have recruited fewer subjects and strengthened the design. This was not done in the current trial due to the long intervention period, which would require at least 4 weeks wash-out period in between the two experimental periods and the possibility of a high drop-out rate. Furthermore, participating in an intervention study may influence eating patterns and lead to underreporting of dietary intake. We instructed the participants to continue eating and activity level as normal during the course of this study, and we did not observe any changes in dietary intake or body weight. However, the estimated energy intake from the diet diaries were lower than anticipated according to weight. It should also be mentioned that body composition was assessed with BIA, which is suggested to be effective in healthy individuals and individuals with a stable water and electrolyte balance. The results should be interpreted with caution in individuals with BMI >34 kg/m^2^ [45], and overestimating of fat-free mass has been observed when using BIA compared to dual-energy x-ray absorptiometry in obese subjects with BMI >35 kg/m^2^ [46]. Since the average BMI in the current study was 32.5 kg/m^2^, we assume that the results from the BIA analysis are reliable. 

No previous publication, to our knowledge, has investigated supplementation with a low dose of a hydrolysate from cod in a population with MetS. To conclude, in this study, we showed that consumption of 4 g of a CPH daily for 8 weeks in individuals with MetS had no effect on fasting or postprandial glucose, insulin levels or GLP-1 levels, lipid profile or body composition. Studies in the future should further evaluate the effect of fish protein hydrolysate on lipid regulation, and preferably with a larger group of participants over a longer time period. Furthermore, the presence of potential bioactive peptide sequences with antidiabetic or lipid-lowering effects in the cod protein hydrolysate and the potential mechanism of effect should be explored further.

## Figures and Tables

**Table 1:** Clinical and physical characteristics of subjects in the cod protein hydrolysate (CPH) group (*n* = 15) and the placebo group (*n* = 15) at screening.

Variables	CPH		Placebo		*p*-Value
	Mean	SD	Mean	SD	
Gender (female/male)	11/4		13/2		0.651
Age, years	52.8	6.26	53.4	6.83	0.804
Anthropometric measurements					
Body weight, kg	96.5	12.8	93.4	12.2	0.509
WC, cm	107.6	9.72	105.7	10.7	0.630
BMI, kg/m^2^	32.7	2.24	32.4	3.25	0.751
Blood pressure (BP)					
Systolic BP, mmHg	136.9	15.9	138.5	15.1	0.756
Diastolic BP, mmHg	88.2	10.1	86.7	6.44	0.702
Glucose metabolism					
Glucose, mmol/L	5.73	0.75	5.63	0.79	0.704
HbA1c, mmol/mol	37.5	4.47	35.7	3.40	0.208
Lipid metabolism					
Total cholesterol, mmol/L	5.8	1.1	5.5	0.8	0.466
HDL cholesterol, mmol/L	1.3	0.3	1.4	0.2	0.493
LDL cholesterol, mmol/L	4.2	0.1	3.9	0.8	0.334
Triacylglycerol, mmol/L	2.10	0.7	2.05	0.6	0.870
Numbers using BP medications	5		9		-
Tobacco users	1		2		-

SD, standard deviation; WC, waist circumference; BMI, body mass index BP, blood pressure; BW, body weight; Hba1c, glycated hemoglobin. Results are presented as the mean ± SD. Groups were compared at baseline using independent samples *t*-test for continuous data and Fisher’s Exact Test for categorical data.

**Table 2:** Estimated energy and macronutrient intake in the cod protein hydrolysate (CPH) group (*n* = 15) and the placebo group (*n* = 15) at baseline and end of study (8 weeks).

Variable	Baseline		8 Weeks		*p*-Value ^2^	*p*-Value ^3^	*p*-Value ^4^
	Mean	SD	Mean	SD			
Energy intake, kcal/day					0.668		0.726
CPH	1882	485	1777	466		0.177	
Placebo	1812	386	1746	410		0.406	
Protein, g/kg BW/day					0.922		0.815
CPH	0.91	0.22	0.85	0.24		0.357	
Placebo	0.91	0.29	0.87	0.23		0.569	
Fat, g/day					0.122		0.396
CPH	84.9	24.4	77.8	24.2		0.128	
Placebo	71.9	20.2	70.2	24.99		0.723	
Carbohydrate, g/day					0.323		0.488
CPH	193.2	46.0	191.7	51.3		0.848	
Placebo	211.1	51.5	198.4	49.3		0.207	
Basal metabolic rate^1^, kcal					0.579		0.743
CPH	1760	334	1774	350		0.211	
Placebo	1696	296	1700	264		0.399	

SD, standard deviation; BW, body weight. ^1^ Derived from the bioimpedance analysis. ^2^ *p*-values comparing groups at baseline are based on independent samples *t*-test. ^3^ *p*-values within groups based on paired sample *t*-test. ^4^ *p*-values comparing change between groups are based on independent samples *t*-test. Data is based on the mean values from a three-day dietary records. Energy and protein content from the supplement was added to the end of study data—CPH group: 4 g protein, 44 kcal; placebo group: 0 g protein, 46.5 kcal. Results are presented as the mean ± SD.

**Table 3:** Anthropometric measurements and results from the bioimpedance analysis (BIA) in the cod protein hydrolysate (CPH) group (*n* = 15) and the placebo group (*n* = 15) at baseline and end of study (8 weeks).

Variable	Baseline		End of Study		*p*-Value^2^	*p*-Value^3^
	Mean	SD	Mean	SD		
Body weight, kg						0.557
CPH	96.02	13.6	96.14	13.8	0.715	
Placebo	93.15	12.7	92.93	12.2	0.694	
BMI, kg/m^2^						0.603
CPH	32.55	2.43	32.59	2.51	0.741	
Placebo	32.27	3.45	32.21	3.49	0.692	
Waist circumference^1^						0.512
CPH	105.5	9.72	108.8	7.25	0.014	
Placebo	105.9	10.7	108.2	9.49	0.040	
Fat mass, %						0.897
CPH	39.91	6.79	39.50	7.12	0.211	
Placebo	40.21	5.37	39.75	5.47	0.105	
Fat mass, kg						0.834
CPH	38.16	7.81	37.82	8.12	0.319	
Placebo	37.46	7.05	37.03	7.46	0.163	
Fat-free mass, kg						0.816
CPH	57.81	11.54	58.33	12.01	0.221	
Placebo	55.71	10.08	55.92	9.02	0.301	
Total body water, kg						0.325
CPH	42.39	8.47	42.45	8.89	0.974	
Placebo	41.29	7.34	40.64	6.77	0.322	

SD, standard deviation; BMI, body mass index. ^1^ For waist circumference, the results are data measured at the screening visit, and presented for only *n* = 13 in the CPH group and *n* = 14 in the placebo group due to missing values. ^2^ P-values within groups are based on paired samples *t*-test. ^3^ P-values comparing change between groups are based on independent samples *t*-test.
